# Foreign Object Debris Detection for Optical Imaging Sensors Based on Random Forest

**DOI:** 10.3390/s22072463

**Published:** 2022-03-23

**Authors:** Ying Jing, Hong Zheng, Chang Lin, Wentao Zheng, Kaihan Dong, Xiaolong Li

**Affiliations:** School of Automation Science and Electrical Engineering, Beihang University, Beijing 100191, China; julyjuly@buaa.edu.cn (H.Z.); linchang@buaa.edu.cn (C.L.); zhengwentao@buaa.edu.cn (W.Z.); by1803103@buaa.edu.cn (K.D.); by1503112@buaa.edu.cn (X.L.)

**Keywords:** foreign object debris, object detection, random forest, optical imaging sensors

## Abstract

In recent years, aviation security has become an important area of concern as foreign object debris (FOD) on the airport pavement has a huge potential risk to aircraft during takeoff and landing. Therefore, accurate detection of FOD is important to ensure aircraft flight safety. This paper proposes a novel method to detect FOD based on random forest. The complexity of information in airfield pavement images and the variability of FOD make FOD features difficult to design manually. To overcome this challenge, this study designs the pixel visual feature (PVF), in which weight and receptive field are determined through learning to obtain the optimal PVF. Then, the framework of random forest employing the optimal PVF to segment FOD is proposed. The effectiveness of the proposed method is demonstrated on the FOD dataset. The results show that compared with the original random forest and the deep learning method of Deeplabv3+, the proposed method is superior in precision and recall for FOD detection. This work aims to improve the accuracy of FOD detection and provide a reference for researchers interested in FOD detection in aviation.

## 1. Introduction

In the field of aviation, foreign object debris (FOD) refers to any substance that is not part of the aircraft and is presented on the airport runways, which could potentially cause damage and may seriously threaten flight safety [1]. FOD mainly includes metal pieces, screws, tire debris, small stones, plastic pipe, and junk, which are hard to find. During aircraft takeoff and landing, FOD could be sucked into the aircraft by the aircraft engine, possibly causing aircraft engine failure. In addition, FOD may puncture the tires of the landing gear of the aircraft. For example, in 2000, the flight crash at Charles De Gaulle Airport in France was caused by a metal strip that fell on the airport runway. In this accident, 113 people lost their lives. It was the most serious air disaster caused by FOD [2]. Since then, FOD detection has been listed as a prime safety measure in the airport. Traditional FOD detection usually adopts a manual screening method, which has poor reliability and low efficiency, and cannot meet the high-safety and fast-throughput requirements of airports. Therefore, many domestic and international airports are actively studying and developing automatic FOD detection systems to reduce FOD risks.

To date, several FOD detection systems have been applied in airports. For example, the Tarsier Radar system developed in the United Kingdom (UK) uses a 94.5 GHz FMCW radar [3]; the FODetect system, developed in Israel, consists of a millimeter wave radar and an optical camera [4]; the FODFinder system developed in the United States (US) contains a millimeter wave radar, GPS, and optical cameras [5]; and the iFerret system developed in Singapore [6] uses optical cameras. These systems are radar-based detection systems, optical-camera-based detection systems, and multi-sensor fusion detection systems. In radar-based detection systems, FOD detection is implemented based on the characteristics of radar returns [7]. The results of radar-based detection systems are favorable for detection of FOD with sizes larger than 5 cm × 5 cm, but poor for objects with smaller size or weak radar returns such as nuts and rubbers [8]. Even though FOD images could be obtained via optical cameras, these images are not employed to detect FOD. If the characteristics of FOD in optical images are utilized to complete FOD detection, then it is of great significance in preventing FOD damage and increasing the utilization rate of airport runways. Although iFerret uses optical images for FOD detection, the detection results are poor for objects smaller than 5 cm × 5 cm.

The advantages of optical-camera-based detection systems are that they can acquire rich information about the environment and can use computer vision technology to detect objects. In recent years, many methods detecting FOD by virtue of optical images have been proposed, which are performed based on traditional computer vision or deep learning. A typical traditional method is to design a segmentation model to find the possible FOD region. For example, an improved region growth algorithm was proposed by Zheng et al. [9] to identify the FOD region on airport runways. A FOD detection algorithm based on image change detection was designed by Xu et al. [10] and Chen et al. [11]. Zhang et al. [12] presented a detection algorithm based on the weighted fuzzy morphology, detecting image edge while reducing noises. An alternative traditional method of detecting FOD focuses on the novel feature descriptors and effective detectors. Hu et al. [13] developed a FOD detection and classification method based on extreme learning machine and several visual features, in which the color, the histograms of oriented gradient (HOG), and the scale-invariant feature transform (SIFT) are extracted and integrated to represent FOD. Aiming at FOD detection, Niu et al. [14] proposed a method combining support vector machine (SVM) and Gabor wavelet, in which Gabor wavelet is adopted to extract effective features to describe FOD, and then SVM is used to classify FOD. Although the above methods are simple, easy to understand, and fast in calculation, their accuracy is limited due to the diversity of FOD type and the interference of airport road surfaces, such as tire marks, marker lines, splice joints, and holes.

In recent years, for computer vision, such as image classification, image segmentation, and object detection, deep-learning-based methods have been widely used [15,16,17]. Inspired by this, many scholars have introduced deep-learning-based methods into FOD detection. Deep-learning-based methods extract high-level semantic information through continuous down-sampling operations, representing the objects more abstractly and accurately. Cao et al. [18] improved the region-based convolutional neural networks for FOD detection. Li et al. [19] and Gao et al. [20] applied YOLOv3 and DeepLabv3+ in FOD detection, respectively. Deep-learning-based methods can automatically extract the features of FOD to reduce human intervention, but they require a huge dataset to learn. Indeed, the reliance on the large variety of unexpected FOD makes it extremely difficult to collect anomalous images for training. Moreover, most neural networks used in object detection or semantic segmentation are designed to detect typical generic objects, such as pedestrians, and they may provide inferior results of FOD detection tasks where a small area is occupied by FOD on the airfield pavement image.

There are also object detection methods based on random forest framework [21,22,23,24]. Typically, this kind of method firstly construct corresponding pixel-level representations with handcraft features. Then, with the obtained features, random forest can be trained to model the distribution of features patterns and inference the class of pixels in the feature space. Shotton et al. [21] proposed semantic texton forests to serve as efficient texton codebooks for image categorization and semantic segmentation. The splitting nodes in semantic texton forests use the raw value of a single pixel, the sum, difference, and absolute difference of a pair of pixels in d × d image patches. To improve the performance of semantic segmentation, Schroff et al. [22] combined the global features, local features, and context-rich information in the splitting nodes of random forest. Being applied in human pose estimation in the depth images by Shotton et al. [23,24], random forest takes the depth comparison between a pair of pixels as the splitting criteria. In random forest, using the simple comparison between a pair of pixels on local image patches for a feature representation has been one of the most popular representation learning methods. It has constraints of using only two points in fixed-size image patches and fixed weights. This does not fully consider the spatial coherence between adjacent pixels, which reflects the structure properties of the object surface and element correlation of the image, similar to the macroscopic observation of human vision.

In this paper, we present a new FOD detection framework based on random forest to detect small-scale FOD with complex background and noise in airfield pavement images. In this way, the FOD detection problem is described as a pixel-wise classification problem. There are two categories, namely foreground and background. The foreground refers to pixels belonging to FOD and the background refers to pixels belonging to airfield pavement. The pixel visual feature (PVF) is firstly designed to reflect the state of pixels and the spatial coherence between pixels. Compared with using several points in fixed-size image patches and fixed weights in random forest, PVF is able to learn weights and size of receptive field by differential evolution algorithm. Then, the random forest employs the learned PVF and calculates the probability of each pixel for each class. Finally, the effectiveness of the proposed method is verified on the FOD dataset collected from airport and annotated at the pixel level. For the capability of the generalization and robustness of random forest [25] and the global optimization ability of differential evolution algorithm [26], FOD could be detected accurately.

The rest of this paper is organized as follows. The overall detection framework is described in Section 2, including the learning process of PVF using differential evolution algorithm, and the training and testing of random forest. In Section 3, the proposed FOD detection system used in this paper is introduced. In Section 4, the effectiveness of the proposed detection method is verified on the FOD dataset. Comparative experiments between this method and other methods are also introduced in this section. Section 5 and Section 6 provide the discussion and conclusions of this paper, respectively.

## 2. Methods

**Figure 1:**
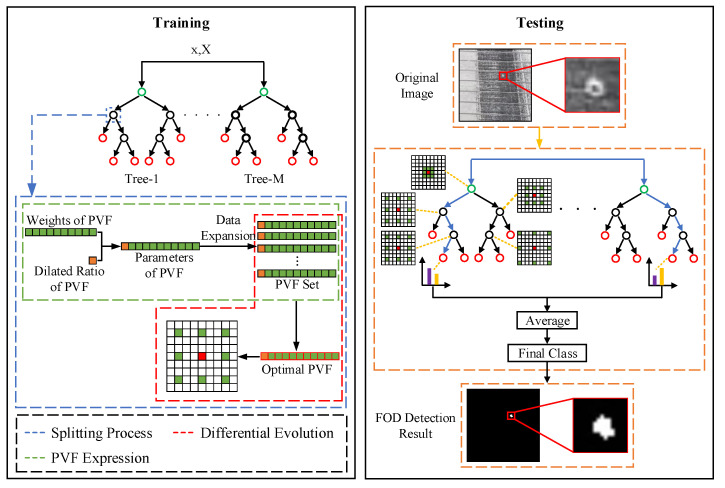
The framework of the proposed foreign object debris (FOD) detection method.

The framework of the proposed method is shown in Figure 1. PVF, that could represent any weight and size of the receptive field, is introduced in Section 2.1. PVF is proposed to learn the optimal features for pixel-wise classification in airfield pavement images during training. In Section 2.2, the random forest uses the PVF. The training process is depicted in Section 2.2.1, and the test process is depicted in Section 2.2.2. In Section 2.3, the location of FOD in airfield pavement images is calculated.

### 2.1. Pixel Visual Feature

Learning the optimal representation is conducive to avoiding unnecessary computations and achieving higher detection accuracy [27]. The typical convolutional neural network (CNN) learns semantic information by filters of various sizes. The recently introduced dilated convolution [28,29] with different dilated ratios can enlarge the receptive field of filters to incorporate more contextual information. Inspired by dilated convolution, we firstly design the dynamic filter whose weights and dilated ratios are learned, not manually defined. Since the spatial coherence between pixels displays the structure properties of the object, we then introduce spatial information to calculate PVF. Finally, the PVF of the pixel *x* consists of two parts, as shown in Equation (1). One is the weighted sum of the pixels in a $\left(2r+1\right)\times \left(2r+1\right)$ patch centered at the pixel *x* using the dynamic filter. The other is the spatial information *V* that is the local variance computed in a $\left(2r+1\right)\times \left(2r+1\right)$ window.

(1) $$f\left(x\right)=\sum_{k}^{}w_{k}X_{x+r\cdot k}+w_{V}V$$

where *x* represents both the input pixel and its spatial location in the image *X*; *k* is the number of pixels used to calculate the PVF of the pixel *x*; x+r·k is the pixel in a $\left(2r+1\right)\times \left(2r+1\right)$ patch centered at the input pixel *x*; $w_{k}$ and *r* are the dynamic filter’s weights and dilated ratio, respectively; $V=\frac{1}{k-1}\sum_{K}^{}{\left(X_{x+r\cdot k}-\bar{X}\right)}^{2}$ and $\bar{X}$ is the mean value of pixels in a $\left(2r+1\right)\times \left(2r+1\right)$ patch; $w_{V}$ is the weight for the spatial information.

**Figure 2:**
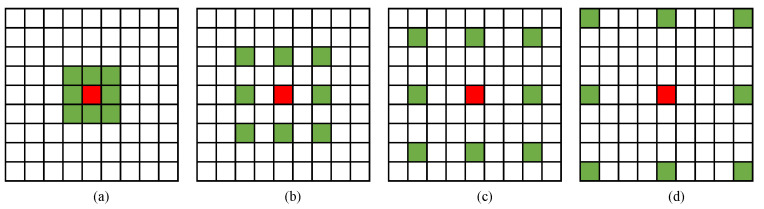
The grid rectangles represent examples of the learned dynamic filters with different weights and receptive fields, the red squares represent the input pixel, and green squares represent the pixels used to calculate the pixel visual feature (PVF). (**a**) *r* = 1. (**b**) *r* = 2. (**c**) *r* = 3. (**d**) *r* = 4.

The weights $w_{k}$ control the influence of the information of the pixel x+r·k, and the dilated ratio controls size of the receptive field of the PVF. As one piece of information in the airport pavement images that can intuitively distinguish different objects in the images, grayscale is adopted to perform FOD detection. The grayscale image can be obtained by color model transformation. Therefore, the PVF consists of 11 parameters, including 10 weights and 1 dilated ratio. All parameters would be learned in the training stage. The learned dynamic filters are described in Figure 2. If any pixel x+r·k is beyond the boundary of the image, then the information $X_{x+r\cdot k}$ is replaced by a constant value, namely 0.

### 2.2. Random Forest

**Figure 3:**
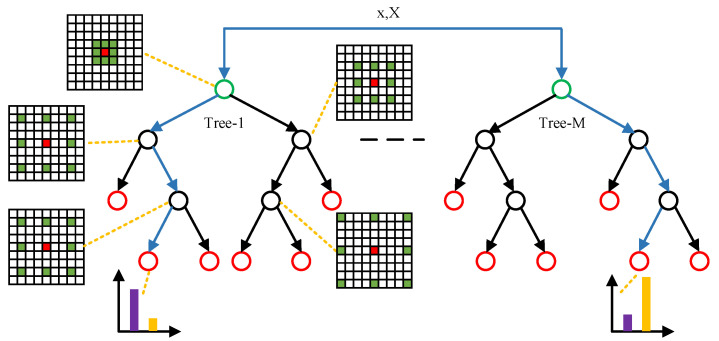
Illustration of the random forest framework. *x* and *X* represent the input pixel and the corresponding grayscale image, respectively. Green, black, and red circles represent root node, splitting nodes, and leaf nodes, respectively. The blue lines indicate the traversal path of the input pixel. The histogram is the probability distribution of the pixel being each class at a leaf node of the decision tree.

Random forest combines multiple decision trees, as shown in Figure 3. Due to the combination of multiple decision trees learned using different data and features, random forest is robust to irregular data and noise [25]. In random forest, each decision tree consists of a root node, splitting nodes, and leaf nodes. The spatial location of the input pixel *x* and the grayscale image *X* are inputs of each decision tree. The input pixel is classified into a child node according to the splitting criteria $f_{n}\left(x\right)⋚\tau$, where $f_{n}\left(x\right)$ is the PVF of the pixel *x* at the node *n*. When the input pixel reaches a leaf node, the classification is terminated. The probability $p\left(c|x\right)$ of being each class *c* at each leaf node is learned in the training process and used in the testing process.

#### 2.2.1. Training

Splitting Criteria: The classification ability of random forest is relevant to its splitting criteria at each node. The splitting process based on the splitting criteria $f_{n}\left(x\right)⋚\tau$ at the node n is defined as follows:

(2) $$\left\{\begin{array}{cc} x\in Q_{1}, & \mathrm{if}\;f_{n}\left(x,X\right)⩽\tau \;\\ x\in Q_{2}, & \mathrm{if}\;f_{n}\left(x,X\right)>\tau \; \end{array}\right.$$

where $Q_{1}$ and $Q_{2}$ are the dataset on the left child node and the right child node of the splitting node *n*, respectively. At a node *n*, the decision boundary is $f_{n}\left(\cdot \right)=\sum_{k}^{}w_{k}X_{x+r\cdot k}+w_{V}V=\tau$, which partitions the instance space into two parts. If the pixel is in region $f\left(\cdot \right)⩽\tau$, then it is classified into the left child node; otherwise, it is classified into the right child node.

We combine learning the optimal PVF with finding the optimal decision boundary through the splitting criteria. Furthermore, for the decision boundary $f\left(\cdot \right)=\tau$, we can eliminate an extra parameter by dividing each side by τ, while ensuring that the data separation remains unchanged, as shown in Equations (3)–(5).

(3) $$\sum_{k}^{}w_{k}X_{x+r\cdot k}+w_{V}V=\tau$$

(4) $$\sum_{k}^{}\frac{w_{k}}{\tau }X_{x+r\cdot k}+\frac{w_{V}}{\tau }V=1$$

(5) $$\sum_{k}^{}w_{k}^{\prime }X_{x+r\cdot k}+w_{V}^{\prime }V=1,\;\mathrm{where}\;w_{k}^{\prime }=\frac{w_{k}}{\tau },w_{V}^{\prime }=\frac{w_{V}}{\tau }\;$$

Therefore, the training algorithm only needs to learn parameters of the dynamic filters while the decision boundary is always 1. In the next section of this paper, weight *w* is used to represent $w^{\prime }$. The simplified splitting process at the node *n* is shown in Equation (6).

(6) $$\left\{\begin{array}{cc} x\in Q_{1}, & \mathrm{if}\;f_{n}\left(x,X\right)⩽1\;\\ x\in Q_{2}, & \mathrm{if}\;f_{n}\left(x,X\right)>1\; \end{array}\right.$$

The following paragraphs present the method of learning the dynamic filters using differential evolution algorithm, the condition for generating a leaf node, and the method of calculating the probability of being each class.

Differential Evolution: As a population-based adaptive global optimization method, differential evolution algorithm uses the rule of survival of the fittest to guide the direction of population update for obtaining the optimal solution. As is depicted in Section 2.1, the parameters to calculate the PVF consists of ten weights and one dilated ratio. The parameters can be regarded as an individual in the population *P*, denoted as $p_{i}$, where 1⩽i⩽N (*N* is the population size). The population can be denoted as $P=\;\left\{p_{1},p_{2},\cdots ,p_{N}\right\}$. The initial values of the population *P* are generated by a random number generator. The parameters affecting the evolutionary process include the scaling factor *F*, the crossover rate Cr, and the maximum number of iterations $g_{max}$. When the loss *L* in Equation (7) no longer decreases at each iteration, or the number of iterations reaches the maximum, and the differential evolution algorithm stops iterating. Differential evolution is implemented to make loss decrease as the iterative number increases, and select the individual with the least loss as the optimal dynamic filter of this iteration. Following this method, the PVF can be evaluated and continuously improved, and the optimal decision boundary is finally identified, achieving the optimal splitting effect.

The training loss *L* is a weighted summation of two losses:

(7) $$L=L_{c}+\lambda L_{R}$$

where $L_{c}$ and $L_{R}$ are classification loss and regularization, respectively, λ the weight for the regularization. Regularization makes the weights *w* evolve in the direction of smaller values.

The classification loss makes the pixels at the nodes belong to the same class. Specifically, we apply the information entropy to measure the purity of the dataset at the nodes. The classification loss is shown below:

(8) $$L_{c}=-\sum_{s}^{}\sum_{c}^{}\frac{Q_{s}}{\sum_{s}^{}Q_{s}}p\left(c|s\right)\mathrm{lg}p\left(c|s\right)$$

where *c* is an index for a class, *s* an index for a child node, including the right child node and the left child node, $Q_{s}$ the number of pixels at the child node *s*, and p(c|s) the proportion of the pixels belonging to the class *c* at the child node *s*.

At each iteration, the update of individual is achieved by sequentially performing three genetic steps, namely mutation, crossover, and selection, as shown in Equations (9)–(11). Mutation refers to sudden and random perturbations added to the genes of the individuals, changing the product of the genes in evolutionary biology. In crossover, the trial individual inherits both the genotype of the target individual and that of the mutant individual, ensuring gene variety and possibly producing high fitness. The selection process ensures that the population evolves in terms of the maximization of the fitness, and never deteriorates.

(9) $$v_{i}^{g}=p_{r_{0}}^{g}+F\left(p_{r_{1}}^{g}-p_{r_{2}}^{g}\right),\;r_{0}\neq r_{1}\neq r_{2}\neq i$$

(10) $$u_{i,j}^{g}=\left\{\begin{array}{cc} v_{i,j}^{g}, & \mathrm{if}\;rand_{i,j}\left(0,1\right)\leq Cr\;\mathrm{or}\;j=j_{rand}\\ p_{i,j}^{g}, & \mathrm{otherwise} \end{array}\right.$$

(11) $$p_{i}^{g+1}=\left\{\begin{array}{cc} u_{i}^{g}, & \mathrm{if}\;L\left(p_{i}^{g}\right)\geq L\left(u_{i}^{g}\right)\\ p_{i}^{g}, & \mathrm{otherwise} \end{array}\right.$$

where $v_{i}^{g}$ is a mutant individual in the *g*-th iteration; $r_{0}$, $r_{1}$, and $r_{2}$ are three randomly generated individual indices; $u_{i}^{g}$ is the trial individual in the *g*-th iteration; rand(0,1) is a uniform random number generator; $j_{rand}$ is a randomly generated parameter index.

After learning the optimal dynamic filter, the pixels at the current node are classified into child nodes. If the conditions for generating a leaf node are not satisfied, then the child node continues to learn the dynamic filter calculating the PVF and generate child nodes; otherwise, the child node will not continue to split and will serve as a leaf node.

Leaf Node: The conditions for generating a leaf node are as follows: (1) the number of training pixels at the node; (2) the probability distribution $p\left(c|x\right)$; (3) the maximum depth of the decision tree. In detail, if the number of remaining training pixels at the node is too small or the probability for a certain class is considerably high, or the current depth of the decision tree reaches the maximum, then the node serves as a leaf node.

When a node is marked as a leaf node, the probability $p\left(c|x\right)$ is calculated according to the number of the pixels belonging to the class *c* at the node, as shown in Equation (12).

(12) $$p\left(c|x\right)=\frac{Q_{L,c}}{\sum_{c}^{}Q_{L,c}}$$

where $Q_{L,c}$ is the number of the pixels belonging to the class *c* at the leaf node *L*.

#### 2.2.2. Testing

The trained random forest is defined as $T=\left\{t_{1},t_{2},\cdots ,t_{m},\cdots ,t_{M}\right\}$. Each pixel *x* in a given image *X* starts from the root node and enters the corresponding child node based on the splitting criteria until it reaches a leaf node using each tree. When it reaches a leaf node *L* using each tree $t_{m}$, the probability $p_{t_{m},L}\left(c|x\right)$ is loaded. Then, the probability from all decision trees are averaged and the class with the highest probability is taken as the final predicted result, as shown in Equation (13).

(13) $$\hat{Y}\left(x\right)=\underset{c}{\arg\max}\frac{1}{M}\sum_{m=1}^{M}p_{t_{m},L}\left(c|x\right)$$

where *M* is the number of decision trees in the random forest *T*.

### 2.3. FOD Location

After identification, the connected domains containing all the FOD-like pixels in the binary image are defined as the predicted FOD. Through marking of the connected domains, the pixels of each disconnected object domain are marked with the same number. After the connected domains are marked, the center coordinates $\left(x_{0},y_{0}\right)$ and the size of each FOD could be calculated. The number of the pixels *z* in the connected domain is used as the size of FOD. The center coordinates of each target are defined as follows:

(14) $$x_{0}=\frac{1}{z}\sum_{(x,y)\in o}^{}x,\;y_{0}=\frac{1}{z}\sum_{(x,y)\in o}^{}y$$

where *o* is a FOD region in the binary image. Finally, the information of the detected FOD are obtained.

## 3. FOD Detection System

**Figure 4:**
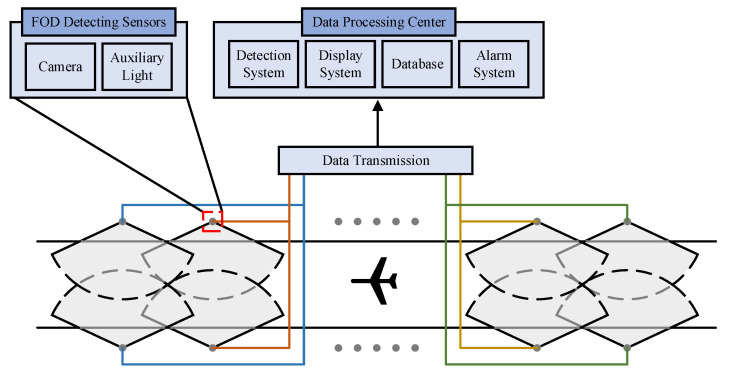
Illustration of FOD detection system.

The FOD detection system in this work is a solution for cost-effective and simple detection of FOD (Figure 4). The system could be installed along both sides of the airport runway. The system is composed of two modules, the FOD-detecting sensors and data processing center. In detail, the FOD-detecting sensors consist of several optical cameras fixed on the pan-tilt, which can provide up, down, left, right, and other azimuth control. The installation cost could be acceptable because the FOD detection sensors can use the power of the runway edge lights. The data processing center receives the optical images captured by the optical cameras, and FOD detection is conducted in this module by the proposed method, providing the information of the detected FOD (such as image and location). According to the information of FOD, the procedures of alarm and clear processing are performed.

Referring to the runway standard of Shahe Airport, the length and width of the runway are 1800 m and 45 m, respectively. The area of the airport runway covered by an optical camera is approximately (200 × 23) $m^{2}$. Therefore, to cover 1800 m of the runway distance, 9 cameras need to be installed. Because one image cannot cover this area, the runway is divided into multiple circular regions. The optical camera focuses on the ring and captures the pavement images along the route. The rotation angle of pan-tilt contains the vertical and horizontal angle. For the same circular region, the vertical angle and the focal length remain unchanged. With the horizontal angle continuing to change, the circular region is scanned by the optical camera. For different circular regions, the vertical angle and the focal length are different. In order to detect nuts with 5 mm in diameter (the smallest FOD sample), the minimum physical size of a pixel is 1 mm.

## 4. Experiments and Analysis

The Section 4.1 introduces the FOD dataset. The evaluation criteria are presented in Section 4.2, and the influence of different parameters is studied in Section 4.3. The effectiveness of the proposed PVF and the differential evolution algorithm is verified in Section 4.4. The comparative experiments of the proposed method with the original random forest [16] and other deep learning methods (Faster R-CNN [30] and Deeplabv3+) [31] are described in Section 4.5.

### 4.1. Dataset

**Figure 5:**
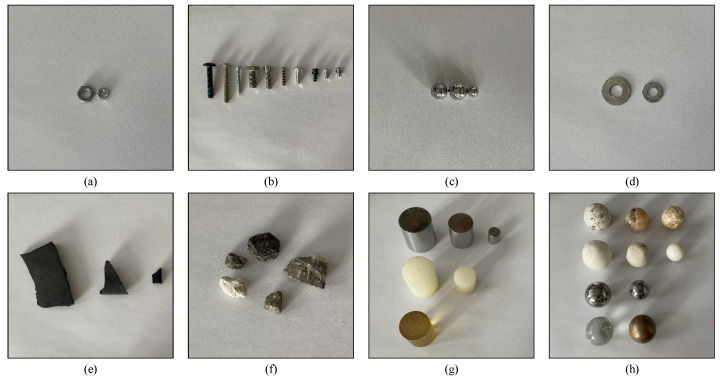
Examples of the FOD samples considered in the FOD dataset: (**a**) nuts; (**b**) screws; (**c**) steel balls; (**d**) gaskets; (**e**) rubber blocks; (**f**) stones; (**g**) cylinders; (**h**) spheres.

The Federal Aviation Administration (FAA) of the United States and the Civil Aviation Administration of China (CDDC) published their advisory circulars, Technical Standards for Airport Foreign Object Debris (FOD) Detection Equipment [1] and the Information Announcement of Airport Runway Foreign Object Debris Equipment [32], respectively, formulating the standards of FOD detection system. Based on the two documents, FOD samples were prepared, including real FOD collected from the airfield pavement and standard samples made by factories. Real FOD samples include nuts, screws, steel balls, gaskets, rubber blocks, and stones, which are major targets for FOD detection, as shown in Figure 5a–f. Standard samples contain spheres and cylinders made of metal, marble, glass, and plastic, as shown in Figure 5g,h. The size of FOD samples is smaller than 5 cm × 5 cm.

**Figure 6:**
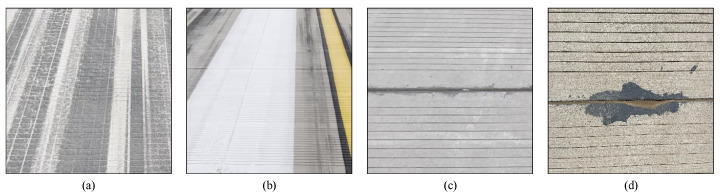
Examples of airfield pavement disturbances: (**a**) tire marks; (**b**) marker lines; (**c**) splice joints; (**d**) holes.

**Figure 7:**
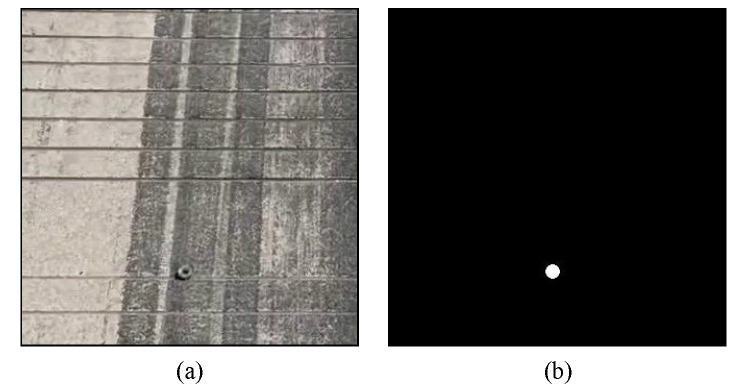
Examples of labeled FOD images: (**a**) original RGB images; (**b**) ground truths.

The FOD dataset was collected by our research group at Shahe Airport in Beijing, China. These images only include the runway pavement, not the sky, grass, or other regions. The FOD dataset contains 1800 RGB images (with a resolution of 1920 × 1080). The dataset is made up of 14 different object categories. Six categories consist of real FOD samples, including nuts, screws, steel balls, gaskets, rubber blocks, and stones. The other 8 categories contain standard FOD samples, including metal spheres, marble spheres, glass spheres, plastic spheres, metal cylinders, marble cylinders, glass cylinders, and plastic cylinders. A single image contains 0 or multiple FODs and there are a 4375 annotation instances. The number of different FOD in the dataset varies from 250 to 350. The area of the FOD samples in the images is less than 15 × 15 pixels on average. Here 70% of the FOD dataset is used as training data, and the other 30% the testing data. To achieve the diversity of FOD dataset, different runway surface disturbances and FOD samples are collected. Runway surface disturbances include tire marks, marker lines, splice joints, holes, and others (Figure 6). The original images were labeled as FOD and ground by labelme software, as shown in Figure 7. The RGB images were used for visual interpretation. The pixel-precise ground truths were manually checked by 5 operators, who would vote for determination in terms of controversial image region.

**Figure 8:**
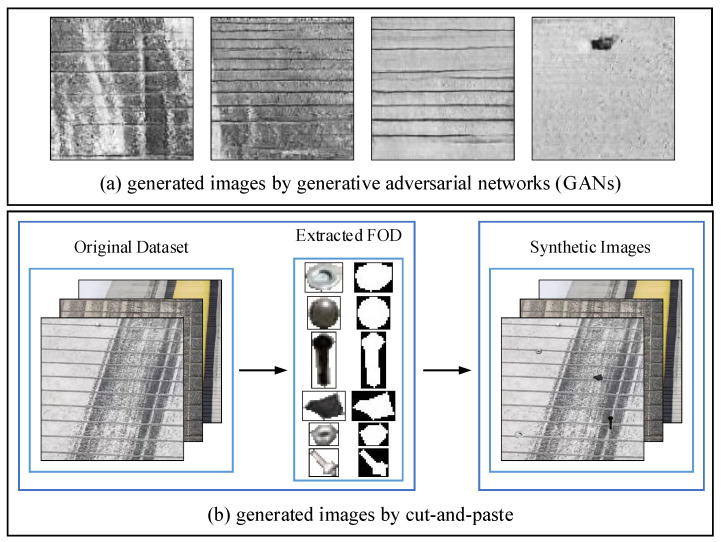
Examples of generated airfield pavement images.

In this paper, for introducing real-world variations and increasing the number of FOD in dataset, the dataset is augmented using two strategies. First of all, we use generative adversarial networks (GANs) [33] to generate high-quality airfield pavement images. The generated images drawn from the generator net after training are shown in Figure 8a. Second, inspired by recent success of synthetic object detection datasets [34] and the fact that many airfield pavement images do not have FOD, a cut-and-paste strategy to synthetically insert FOD into airfield pavement images for data augmentation is employed. As shown in Figure 8b, we first cut numerous FOD objects from the original FOD dataset, and generate synthetic airfield pavement images by inserting FOD in the original images. In this paper, OFOD is used to represent the original FOD dataset and SFOD is used to represent the set of the synthetic FOD dataset and the original FOD dataset.

### 4.2. Evaluation Criteria

To evaluate the performance of the proposed FOD detection method, two benchmark metrics, i.e., precision and recall, are employed. In this study, the proposed FOD detection method acts directly on pixels and infers the category of each pixel. There are mainly two results, FOD and background, of which FOD is denoted by 1 and background by 0. To illustrate the calculation methods of the 2 metrics, 3 types of detection results are defined: (1) $n_{11}$ is the number of pixels correctly detected as FOD; (2) $n_{01}$ is the number of pixels incorrectly detected as FOD; and (3) $n_{10}$ is the number of pixels incorrectly detected as background.

According to $n_{11}$, $n_{01}$, and $n_{10}$, precision and recall are calculated by Equations (15) and (16). *Precision* indicates the proportion of real FOD pixels in the FOD pixels predicted by the detection method. *Recall* indicates the proportion of FOD pixels predicted correctly by the detection method in the real FOD pixels.

(15) $$Precision=\frac{n_{11}}{n_{11}+n_{01}}$$

(16) $$Recall=\frac{n_{11}}{n_{11}+n_{10}}$$

### 4.3. Experimental Setup

The influence of different parameters of the proposed method on FOD detection results is demonstrated in this section. According to the experimental results, the experimental setup is described. The parameters come from two parts, differential evolution algorithm and decision trees. The parameters affecting the evolutionary process include the population size, the maximum number of iterations, the scaling factor, and the crossover rate. The parameters of the decision trees include the number of decision trees and the maximum depth of decision trees.

**Figure 9:**
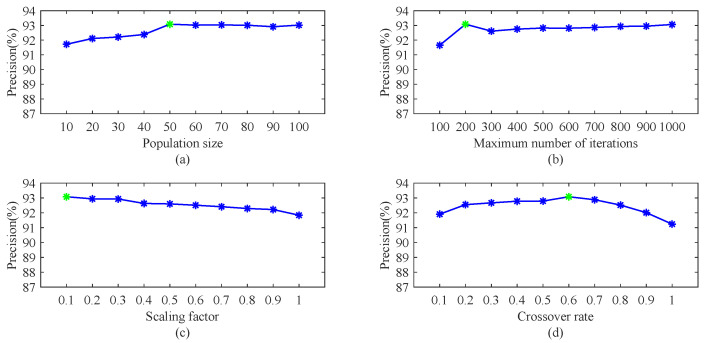
Precision vs. parameters in differential evolution algorithm.

**Figure 10:**
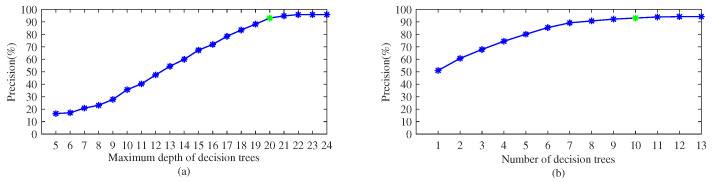
Precision vs. parameters of decision trees.

The detection process including training and testing is repeated five times and the training and testing dataset are reselected for each detection process. All the reported results are averaged over the five experiments. The experimental method is control variable method. First, all parameters are set to their default value, indicated by green marks in Figure 9 and Figure 10, respectively. Then, in each experiment, only a parameter value is changed while fixing the remaining five parameters’ values. Using the method, we compared and analyzed the impact of the main parameters on the performance of the proposed method.

Figure 9a shows the performance of the proposed method when the population size changes. The performance is improved with respect to the population size. However, when the population size is greater than 50, its impact on the detection performance becomes negligible and can even be ignored. Figure 9b uncovers the influence of the maximum number of iterations. When the maximum number of iterations is less than 200, the performance is significantly improved, yet when the number of iterations is more than 200, the improvement of the performance is not obvious. Figure 9c presents influence of the scaling factor. It can be observed that the detection performance deteriorates with the enlargement of scaling factor. Figure 9d displays the influence of the crossover rate. It can be seen that the procedure is sensitive to the crossover rate, and the crossover rate with 0.6 performs the best.

Figure 10a shows the relations between detection performance and the maximum depth of decision trees. It can be observed that the maximum depth of decision trees is nearly proportional to the performance when the maximum depth is less than 22. The improvement is not obvious when the maximum depth is greater than 22. The possible explanations that a large number of the maximum depth leads to over-fitting. Figure 10b shows the relations between detection performance and the number of decision trees. It can be observed that the performance becomes better when the number of decision trees increases, though eventually they would level out. In detail, when the number of decision trees is less than 7, precision can be increased by about 7% by adding a decision tree. However, when the number of decision trees is greater than 7, precision can only be increased by about 1% by adding a decision tree. This proves that when the number of decision trees exceeds a certain value, increase in the number of decision trees only has a limited improvement effect on the performance.

The experimental results reveal that compared with the number of decision trees, the maximum depth of decision trees has a greater impact on the performance of the proposed method. The reason may be that the depth of decision trees directly affects the classification ability of the proposed method, while the number of decision trees merely affects the robustness of the proposed method to the noise of the data.

Based on the above experiments, the following parameters are used unless stated otherwise. The population size is set as 50, the maximum number of iterations 200, the scaling factor 0.1, and the crossover rate 0.6. In consideration of the increased computational complexity and memory usage resulted from the increased maximum depth and number of decision trees, the maximum depth of decision trees is set as 20 and the number of decision trees 10. The conditions for generating leaf nodes are that the probability of being a class is 0.99% and the amount of the remaining data is 0.01% of the total training data.

### 4.4. Validity of the Proposed Method

This section demonstrates the effectiveness of the proposed PVF and the differential evolution algorithm compared with the typical convolution kernel and random generation in Table 1. The performance of the random forest based on PVF outperforms the random forest based on 3 × 3 convolution kernel and the former has smaller number of nodes. The analysis shows that the PVF, learned by differential evolution algorithm, outperforms that learned through random generation. In terms of the spatial information, the detection result with PVF integrating the spatial information is better than that of without integrating the spatial information. It can be concluded that the random forest based on the proposed PVF and the differential evolution algorithm can learn more optimal representations.

### 4.5. Comparative Experiments

This section compares the proposed method with the original random forest and deep learning methods (Deeplabv3+ and Faster R-CNN) on FOD dataset using a machine with Intel i9-9940 CPU with 3.30 GHz, 32 GB RAM, and NVIDIA GeForce RTX 2080 Ti GPU.

**Figure 11:**
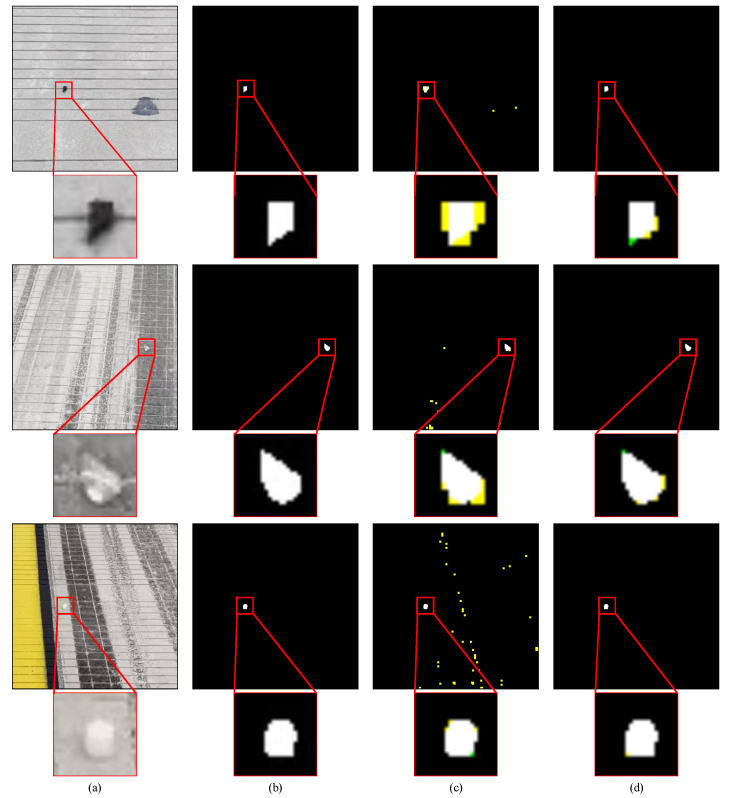
Qualitative comparison between the proposed method and the original random forest: (**a**) original RGB images; (**b**) ground truths; (**c**) results of the original random forest; (**d**) results of the proposed method. The white region indicates the pixels that are correctly detected as FOD, the black indicates the pixels that are correctly detected as background, the yellow indicates the pixels that are incorrectly detected as FOD, and the green indicates the pixels that are incorrectly detected as background.

The proposed method and the original random forest were trained on the OFOD dataset, which includes only the original FOD data and is defined in Section 4.1. The precision and recall of the original random forest are about 76.18% and 92.93%, respectively. The detection precision of the proposed method is about 17.69% higher than that of the original random forest. Figure 11 present the qualitative results of the proposed method and the original random forest. In Figure 11, the image in the first row contains a piece of rubber block, the second row a gravel, and the third row a white cylinder made of plastic. According to Figure 11, the original random forest raised many false alarms in the regions with airfield pavement disturbances, such as tire marks and holes. This proves that the proposed method can better suppress the interference and noise on the airfield pavement and segment foreign objects from the airfield pavement than the original random forest.

**Figure 12:**
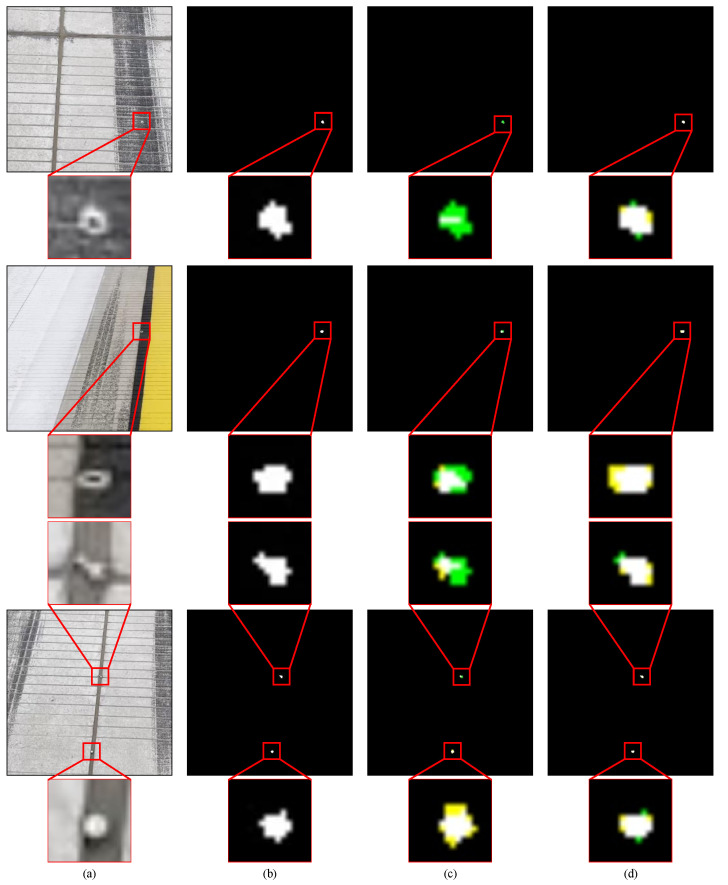
Qualitative comparison between the proposed method and Deeplabv3+: (**a**) original RGB images; (**b**) ground truths; (**c**) results of Deeplabv3+; (**d**) results of the proposed method. The white region indicates the pixels that are correctly detected as FOD, the black indicates the pixels that are correctly detected as background, the yellow indicates the pixels that are incorrectly detected as FOD, and the green indicates the pixels that are incorrectly detected as background.

**Figure 13:**
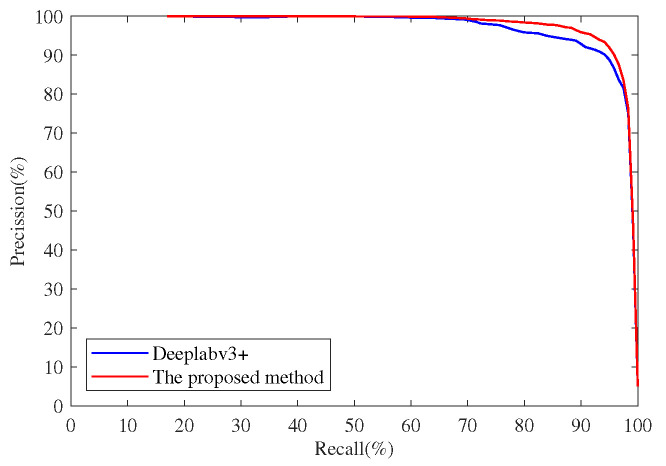
Comparison of Precision-Recall (PR) curves of different methods.

Table 2 and Figure 12 show the comparison of the proposed method with Deeplabv3+, trained on the SFOD dataset. In Figure 12, there is a nut in the first row, a gasket in the second row, and a screw and a steel ball in the third row. Compared with Deeplabv3+, the precision of the proposed method is increased by 0.91% and the recall 3.06%. The results in Table 2 demonstrate that the proposed method has a better detection effect on small-scale FOD than Deeplabv3+. The Precision-Recall (PR) curves of the two methods are shown in Figure 13. The results in Figure 12 demonstrate that the proposed method and Deeplabv3+ are all robust to the interference and noise of airfield pavement. The detailed images in Figure 12 show the FOD edge detection results of the two methods. Deeplabv3+ records many omission errors and false alarms in FOD edge detection. In conclusion, compared with Deeplabv3+, the proposed method performs better in control of omission errors and false alarms, and in the display of FOD edge details.

We discuss the possible explanations for why the precision of the deep learning method is limited compared with the proposed method. Some FOD is quite small, such as screws, nuts, and steel balls that fall from the aircraft. Additionally, since cameras are generally installed with a large view from a long distance, the pixels of FOD in image are less than 15 × 15 pixels on average. Therefore, the task of FOD detection is essentially small objects detection. Although deep learning methods are widely used, the problem of small-scale FOD detection is still not fully resolved [35]. First, small objects often reveal extremely limited appearance information, which increases difficulty in learning discriminative features. Second, with the continuous decline of the resolution of feature map in deep neural networks, the area of small objects is also gradually decreasing and even the spatial information on the feature map is lost. Finally, since deep learning methods often predict pixel-level labels on a low-resolution feature map, e.g., 1/16-th of the input for Deeplabv3+, or 28 × 28 mask for Mask-RCNN [36], boundary recovery has been a challenge for deep neural networks in image segmentation [37].

**Figure 14:**
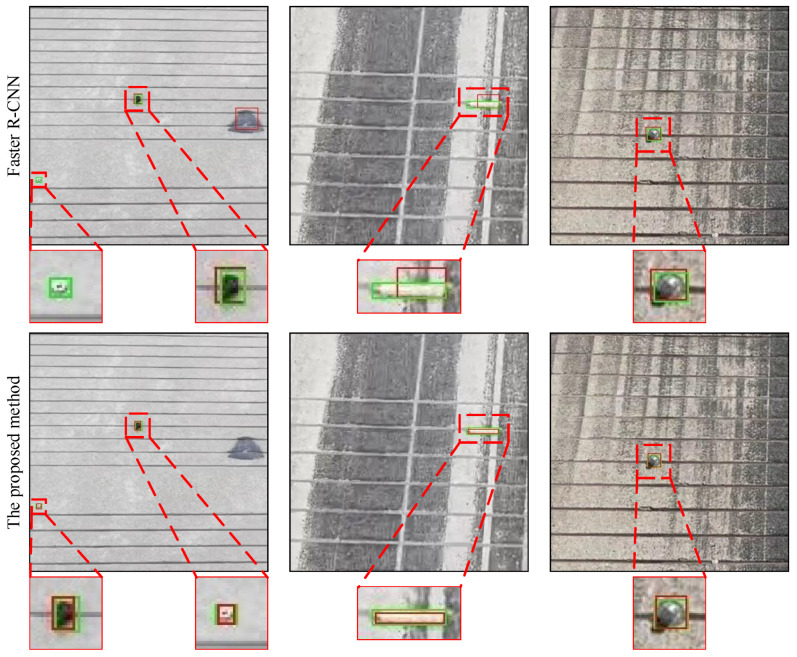
Qualitative comparison between the proposed method and Faster R-CNN. The green solid boxes indicate the ground truths while the red solid boxes refer to the detected objects.

Table 3 and Figure 14 show the comparison of the proposed method with Faster R-CNN, trained on the SFOD dataset. In Figure 14, there is a nut and a rubber block in the first column, a copper pillar in the second column, and a metal sphere in the third column. To compare the performance of two methods, the mean average precision (mAP) is calculated. The proposed method achieves 93.47% mAP and is higher 3.3% than Faster R-CNN, which means that the proposed method is effective and robust for small-scale FOD detection. This is because the proposed method classifies pixels by learning their optimal representations without down-sampling, which retains all information of FOD. However, Faster R-CNN extracts high-level feature representations by continuous down-sampling operations, damaging the information of small-scale FOD, whose poor-quality appearance and structure also increase the difficulty of learning rich features [38].

## 5. Discussion

In this study, the current research on FOD detection method is investigated. It can be found that there are shortcomings in existing detection algorithms based on optical images, i.e., the poor result on small FOD and not considering interference of airport road surface. Consequently, an object detection method for small FOD under complex background is proposed. Random forest and differential evolution are introduced into FOD detection tasks. The concept of PVF is proposed, which learns the optimal feature to replace the manual design process. The process of learning the optimal PVF is combined with finding the optimal decision boundary to split data in nodes, which are divided into two classes: FOD pixel data and non-FOD pixel data. In this way, the problem of FOD detection is transformed into the problem of pixel classification, achieving pixel-level FOD detection. In the process of decision tree construction, the optimal splitting results are scored by the information entropy and the differential evolution.

For random forest, it is necessary to provide features of the data to use for segmentation in the process of decision tree construction. Inspired by the use of convolutional kernels to learn features in deep neural networks, the convolutional kernels are introduced to learn the state and spatial correlation of pixels. According to the experimental results in Section 4.4, due to the diversity of FOD type and the interference of airport road surface, the PVF of pixels cannot be calculated by 3 × 3 convolution filters with random weights and fixed receptive field. In the proposed method, the weights and the size of receptive field are able to learned using differential evolution at the same time, so as to distinguish between FOD pixels and background pixels accurately. By comparison, the detection accuracy of the proposed method for FOD is better than that of other methods.

The FOD dataset is collected to verify the effectiveness of the method. Although the types of FOD are unknown in advance, we selected many object categories based on guidance from related research by FAA and CDDC. Currently, the FOD dataset has included 14 object categories and 4375 annotation instances. Varying object categories ensures the complexity of the FOD dataset. However, there is still a limitation. The FOD dataset does not consider the variability of the real world, which includes different light and weather conditions. In future research, we will continue to expand the FOD dataset from two aspects: collecting different FOD samples and collecting images under different light and weather conditions to ensure accurate simulation of airport environments.

## 6. Conclusions

To improve the detection accuracy of small-scale FOD in a complex background, this study proposes a novel FOD detection framework based on random forest, which employs representation PVF to accurately segment FOD regions and effectively suppresses background interference in airfield pavement images. PVF is designed to learn the optimal representation to achieve better detection performance. The random forest is chosen to achieve higher accuracy for small-scale FOD detection. The deep combination of random forest and PVF endows the proposed method with higher robustness and generalization for FOD detection. In the experiment section, compared with the original random forest, the proposed method remains undisturbed by airfield pavement disturbances. Compared with Deeplabv3+, the proposed method exhibits a better performance in recall and precision.

Future research will employ image pyramids in feature representation to promote the performance of FOD detection. Furthermore, an enlarged FOD dataset will be built, covering different lighting conditions, such as full sunlight and cloudy weather, to evaluate our proposed detection algorithm in future research.

## Figures and Tables

**Table 1:** Analysis of the proposed representation PVF.

Method		Number of Nodes	Precision	Recall
Representation	Optimization			
Convolution Kernel (3 × 3)	Differential Evolution	36,585	78.58%	79.20%
PVF	Random Generation	26,749	84.68%	84.98%
PVF	Differential Evolution	23,998	93.08%	94.21%
PVF (+Spatial Information)	Differential Evolution	22,635	93.87%	95.01%

**Table 2:** Comparison between the proposed method and Deeplabv3+.

Method	Precision	Recall
Deeplabv3+	93.97%	92.37%
The proposed method	94.88%	95.43%

**Table 3:** Comparison between the proposed method and Faster R-CNN.

Method	mAP
Faster R-CNN	90.17%
The proposed method	93.47%

## Data Availability

The data presented in this study are available on request from the corresponding author. The data are not publicly available due to privacy.
